# The relative test performance characteristics of two commercial assays for the detection of *Mycobacterium tuberculosis *complex in paraffin-fixed human biopsy specimens

**DOI:** 10.1186/1746-1596-3-37

**Published:** 2008-09-08

**Authors:** Steven J Drews, AliReza Eshaghi, Daria Pyskir, Pam Chedore, Ernesto Lombos, George Broukhanski, Rachel Higgins, David N Fisman, Joanne Blair, Frances Jamieson

**Affiliations:** 1 Public Health Laboratories Branch, Ministry of Health and Long-Term Care, Toronto, Ontario, Canada; 2 Department of Microbiology, Mount Sinai Hospital, Toronto, Ontario, Canada; 3 Hospital for Sick Children, Toronto, Ontario, Canada; 4 Department of Laboratory Medicine and Pathobiology, University of Toronto, Toronto, Ontario, Canada

## Abstract

The Seeplex™ TB Detection-2 assay (Rockville, MD) is a nested endpoint PCR for the *Mycobacterium tuberculosis *complex (MTBC) targets IS*6110 *and MPB64 that utilizes dual priming oligonucleotide technology. When used to detect the presence of MTBC DNA in formalin-fixed paraffin-embedded tissue specimens, the sensitivity and specificity of this assay is equivalent to a labor-intensive traditional endpoint PCR assay and is more sensitive than a commercial real-time PCR assay.

## Findings

Since December of 2000, the Central Public Health Laboratory (CPHL) of the Ontario Public Health Laboratories, Ministry of Health and Long-Term Care has utilized an in-house nucleic acid amplification testing followed by Sanger sequencing for the detection of *Mycobacterium tuberculosis *complex (MTBC) in formalin-fixed paraffin-imbedded tissue specimens. Although this methodology has allowed for the characterization of approximately 250 tissue specimens, it is labor intensive and requires the use of an expensive sequencing protocol to ensure assay specificity. During this time, several commercial kits and other published methodologies have become available which allow for the molecular diagnosis of MTBC in biopsy specimens and utilize specific mechanisms to ensure assay specificity. The targets of these assays include IS*6110 *[1] and MPB64 [2] using either traditional endpoint PCR or real-time PCR.

Traditional endpoint PCR is often thought to lack the analytical sensitivity and specificity when compared to real-time PCR technology [3,4]. Although real-time PCR may offer increased sensitivity and specificity, several articles have described failures of real-time PCR assays to detect MTBC due to slight changes in primer and probe sequences [5]. When using end-point PCR reactions, specificity can be increased through the use of sequencing technology or restriction enzyme analysis of PCR products. Dual-priming oligonucleotide technology (DPO) has also been recently described in the literature as a method that increases both the sensitivity and specificity of endpoint PCR reactions [6]. As a result, this methodology provides a means of confirming sequences without the requiring real-time PCR or extensive processing of PCR products for use in sequencing reactions or restriction enzyme analysis [6]. Recently, a commercial DPO-based assay (Seegene, Rockville, MD) utilizing nested PCR for MTBC specific target genes IS*6110 *and MPB64, became available.

This manuscript describes the verification of the Seeplex™ TB Detection-2 assay (Seegene, Rockville, MD) and the artus^® ^M. *tuberculosis *TM assay (Qiagen, Mississauga, ON, Canada) on formalin-fixed paraffin-embedded tissue specimens that had been previously tested using the in-house assay, and an analysis of the turn-around-time (TAT) and workflow required for performance of the assays [1].

Purified *M. tuberculosis *H37Rv DNA was used as standard for analysis of limit of detection for multiple methods. Theoretical limits of detection for each methodology were determined using serial 10-fold dilutions of *M. tuberculosis *H37Rv DNA in PCR grade water. The theoretical number of copies of *M. tuberculosis *H37Rv DNA was calculated according to a generally accepted conversion formula.

Formalin-fixed paraffin-embedded tissue sample blocks are submitted to the CPHL with a copy of the corresponding histopathology report. Depending of the size of the tissue block, either the entire tissue, or portions of the tissue block (determined histopathologically) were de-paraffinized and DNA extraction prepared using QiaAMP spin column (QIAGEN, Mississauga, Ontario) protocol [7]. DNA was frozen at -80°C until further use in PCR protocols.

The in-house reference method was a modification of a previously published method [1] and has been used as a diagnostic tool at the authors' facility since December 2000. Briefly, 5 μl of extracted DNA were used in a non-nested PCR reaction utilizing the DNA minikit (QIAGEN, Mississauga, Ontario) procedure, 0.3 μM primer IS-1 (5'-CCT GCG AGC GTA GGC GTC GG-3') and 0.3 μM IS-2 (5'-CTC GTC CAG CGC CGC TTC GG-3') using the PCR4 reaction (one 4 minute denaturation step at 95°; 45 cycles of 30 seconds at 95°C, 30 seconds at 63°C, 30 seconds at 72°C; and a 7 minute extension at 72°C) [1]. In addition, amplicons of the appropriate molecular mass were identified by EtBr staining of a 1% agarose gel electrophoresis and due to the presence of some non-specific banding, amplicons of the appropriate size were confirmed by Sanger sequence analysis using the ABI BigDye^® ^Terminator v3.1 Cycle Sequencing Kit on an ABI 3100 genetic analyzer (Applied Biosystems, Foster City, CA). IS*6110 *sequences were compared to accessioned sequences using the Basic Local Alignment Search Tool (BLAST) and the NCBI database . Sequences were considered to be identified as IS*6110 *if they shared the highest identity in the BLAST search. Amplicons were also restricted by 10 units of *Sal*I (New England Biolabs, Ipswich, MA) at 37°C for 3 hours. Digested amplicons were analyzed by 3% agarose gel electrophoresis and EtBr staining. DNA from the control strain MT14323 was spiked into the clinical specimens that had negative results to determine if this was a result of PCR inhibition [8].

To control for DNA extraction, all specimens that were negative for IS*6110 *by the uninhibited reference in-house PCR method were assayed for the human gene *gapdh *using a commercial *gapdh *assay kit and an ABI 7900 HT thermocycler (Applied Biosystems, Foster City, CA).

Between May 2007 and January 2008, DNA from specimens was first extracted from formalin-fixed paraffin-embedded tissue blocks and tested against the in-house reference method. DNA from specimens that were determined to be either positive or negative by the reference method were frozen at -80°C until further analysis. It should be noted that DNA from specimens that were positive by the in-house reference method were also confirmed to be positive by sequencing of IS*6110 *product fragments.

DNA extracted from the formalin-fixed paraffin-embedded tissue blocks from 21 IS*6110*- positive and 35 IS*6110*-negative specimens by the reference method was concurrently analyzed by two commercially available methods, the Seeplex™ TB Detection-2 assay (Seegene, Rockville, MD) and the artus^® ^*M. tuberculosis *TM PCR assay (for use with the ABI 7900 HT as per manufacturer's protocols). The amount of DNA used varied as per the kit/assay manufacturer's instructions. Inputs for each assay used were as follows: Seeplex™ TB detection-2 assay utilized 3 μl for initial and nested PCR reactions; artus^® ^*M. tuberculosis *TM PCR kit, 10 μl. Results were compared to the in-house assay results.

The Seeplex™ TB Detection-2 assay was carried out using an iCycler (Bio-Rad, Hercules, CA) and 1% agarose gel electrophoresis and EtBr staining for amplicon detection. This assay contains an integrated control for PCR inhibition. The Seeplex™ TB Detection-2 is a nested PCR involving a first run PCR (1 at cycle 94°C for 15 minutes; 35 cycles at 94°C for 30 seconds, 60°C for 30 seconds, 72°C for 30 seconds; 1 cycle at 72°C for 5 minutes) and a nested PCR (1 cycle at 94°C, 15 minutes; 25 cycles at 94°C for 30 seconds, 62°C for 30 seconds, 72°C 30 seconds; 1 cycle at 72°C for 5 minutes). The artus^® ^*M. tuberculosis *TM PCR kit followed the assay protocol with the following protocol: 1 cycle 95°C for 2 minutes; 45 cycles at 95°C for 15 seconds; and 64°C for 1 minute. In the event of internal control inhibition, total nucleic acids from specimens were diluted 1/10 and 1/100 in PCR grade dH_2_0 and re-tested as per each assay protocol.

The time required to complete different methods was compared by a retrospective review of work flow from laboratory records. Chi-squared analysis was carried out using GraphPad Prism software (GraphPad Software Inc., El Camino Real, CA). Probit regression analysis was carried out using SPSS 15 software (SPSS Inc., Chicago, IL).

Thirty-five specimens that were negative by the in-house reference method were tested by the two commercial methods. All specimens demonstrated successful DNA extraction as all specimens were *gapdh*-positive. The proportions and numbers of formalin-fixed paraffin-embedded tissues per specimen type were as follows: pulmonary (46%, 16/35), lymphatic (23%, 8/35), gastrointestinal (14%, 5/35), musculoskeletal (6%, 2/35), central nervous system (6%, 2/35), endothelial (3%, 1/35), and integument (3%, 1/35).

Twenty-one specimens that were positive by the in-house reference method were tested by the two commercial methods. All specimens demonstrated successful DNA extraction as all specimens were *gapdh*-positive. The proportions and numbers of formalin-fixed paraffin-embedded tissues per specimen type were as follows: lymphatic (33%, 7/21), pulmonary (24%, 5/21), genitourinary (14%, 3/21), musculoskeletal (14%, 3/21), central nervous system (5%, 1/21), gastrointestinal (5%, 1/21), and integument (5%, 1/21). There was no significant difference between proportion of specimen types in positive and negative specimens when analyzed for pulmonary, lymphatic and a combination of all other specimens (χ^2 ^= 2.697, df = 2, p = 2.697).

Table 1 details the performance characteristics of each assay to identify MTBC in formalin-fixed paraffin-embedded human tissue specimens. The in-house IS*6110 *PCR, sequencing, and restriction analysis were used as the reference standard for specificity and sensitivity analyses. Inhibition of PCR occurred in 10/35 (29%) reference test negative specimens tested by the artus^® ^*M. tuberculosis *MTB assay. Specimens were re-tested after dilution of extracted nucleic acid in 1/10 dH_2_0, with all but one specimen being resolved. The one unresolved specimen remained inhibited even after a 1/100 dilution of extracted DNA in dH_2_0. Removing the inhibited negative specimen from analysis, the specificity of the artus^® ^*M. tuberculosis *TM PCR kit was 100% (34/34). In contrast, 6% (2/35) of reference test negative specimens were initially inhibited when the Seeplex™ TB Detection-2 kit was used on specimens. It should be noted that both of these specimens were also inhibited when tested by the artus^® ^*M. tuberculosis *MTB assay. All of these inhibited specimens were resolved by 1/10 dilution of the extracted nucleic acid in dH_2_0. Inhibition of PCR reactions varied between assays. The specificity of the Seeplex™ TB Detection-2 kit when compared to IS*6110 *PCR, sequencing and restriction analysis was (35/35) 100%.

**Table 1:** Relative test performance of two commercia1 assays for MTB complex detection in formalin-fixed parafinized biopsy specimens.

**Result**	**Methodology**	
	**artus^® ^*M. tuberculosis *TM PCR**	**Seeplex™ TB Detection-2 assay**
% (#) inhibited negative* specimens (n = 35)	29 (10)	6 (2)
% (#) total negative* specimens with resolved inhibition (n = 35)	97 (34)	100 (35)
% (#) inhibited positive* specimens (n = 21)	24 (5)	0 (0)
% (#) total positive* specimens with resolved inhibition (n = 21)	76 (16)	95 (20)

*as determined by reference method (IS*6110 *PCR, sequence analysis, and restriction enzyme analysis)

Inhibition of PCR occurred in 5/21 (24%) of reference test positive specimens using the artus^® ^*M. tuberculosis *TM PCR kit. Inhibition could not be overcome by 1/10 and 1/100 dilution of nucleic acid in dH_2_0. Although inhibition could be resolved in four of five specimens by dilution of nucleic acid in 1/10 dH_2_0, these dilutions failed to identify MTBC targets in these specimens. Thus, due to assay inhibition and attempts to overcome this inhibition, only 76% (16/21) of positive specimens were detected by the artus^® ^*M. tuberculosis *TM PCR assay. When inhibited specimens that failed to resolve were discarded from analysis, the sensitivity of the artus^® ^*M. tuberculosis *TM PCR kit was 80% (16/20). In contrast, none of the positive specimens were inhibited when the Seeplex™ TB Detection-2 kit was utilized. Although the sensitivity of the Seeplex™ TB Detection-2 kit was 95% (20/21) (Table 1) the single discordant specimen was only positive by the reference method, and was also unresolved using the artus^® ^*M. tuberculosis *TM PCR kit.

The limit of detection of *M. tuberculosis *H37Rv as determined by Probit regression (95% probability) for each methodology was estimated to be; Seeplex™ TB Detection-2 assay (0.4 genome copies per reaction), artus^® ^*M. tuberculosis *TM PCR assay (10 genome copies per reaction) and the in-house assay (0.2 genome copies per reaction). Based on an average 7.5 h workday, specimen extraction and the in-house reference assay had an approximate TAT of 72 hours. Including specimen extraction time, the approximate TAT for the Seeplex™ TB Detection-2 kit was 6 hours while the average TAT for the artus^® ^*M. tuberculosis *TM PCR was 4 hours. The estimated average cost per specimen by the three methods in Canadian dollars was as follows: artus^® ^*M. tuberculosis *TM PCR at $40, Seeplex™ TB Detection-2 kit at $15, and the in-house reference assay at $5.

The artus^® ^*M. tuberculosis *TM PCR kit is a closed, real-time system assay with less requirement for manual manipulation, but had problems with inhibition and decreased sensitivity when compared to the in-house reference method. The poor performance of this assay when compared to the Seeplex™ TB Detection-2 assay may be a product of the interaction between PCR kit, specimen and extraction method. The sensitivity of the artus^® ^*M. tuberculosis *TM PCR kit may be negatively affected by the higher limit of detection (10 genome copies per reaction) when compared to 0.4 genome copies per reaction for the nested Seeplex™ TB Detection-2 assay and 0.2 genome copies per reaction for the non-nested reference method. Thus, in some specimens, dilutions of DNA used to overcome the inhibitory nature of the matrix used in this study (e.g. in formalin-fixed paraffin-imbedded tissue specimens) may have forced the concentration of MTBC DNA below the limit of detection of the assay. It is also possible that some factors such as small changes in real-time primer and probe design may also have an effect on the relative poor performance of the real-time PCR kit. Given these multiple effects, the authors believe that the artus^® ^M. *tuberculosis *TM PCR kit may have improved test characteristics with less complex specimens (e.g. fresh tissue) or an extraction method capable of removing assay inhibitors.

In conclusion, the Seeplex™ TB Detection-2 kit offers equivalent sensitivity and specificity to a more labor-intensive endpoint PCR method and improved sensitivity when compared to a commercial real-time product. The presence of an internal control directly within this assay allows for the determination of PCR inhibition when testing these potentially inhibited specimens.

## Abbreviations

DNA: Deoxyribonucleic Acid; EtBr: Ethidium Bromide; PCR: Polymerae Chain Reaction; TB: Tuberculosis.

## Competing interests

The authors declare that they have no competing interests.

## Authors' contributions

SJD planned experiments, coordinated experiments and wrote article, AE undertook sequencing and enzyme analysis, DP undertook primary PCR and enzyme analysis, PC undertook specimen coordination, GB assisted in assay optimization, EL undertook PCR for MTB and extraction controls, RH designed enzyme analysis steps, DNF assisted with planning and conceptualization, JB assisted with study design, FJ coordinated and planned the experiments and helped write the article.
